# A rare case of Gardner syndrome in an African adult male: A case report

**DOI:** 10.1002/ccr3.8735

**Published:** 2024-04-03

**Authors:** Olusegun I. Olaopa, Adedamola A. Dada, Oluwafunmilayo Y. Soneye, Oluwadamilare Iyapo, Taofeek A. Akinniyi, Akinyele O. Adisa, Kehinde K. Kanmodi, Adedolapo O. Olaopa, Christian I. Emeka, Imudia D. Ehanire, Modupe O. Coker

**Affiliations:** ^1^ Department of Dental Services Federal Medical Centre, Ebute‐Metta Lagos Lagos State Nigeria; ^2^ Department of Surgery Federal Medical Centre, Ebute‐Metta Lagos Lagos State Nigeria; ^3^ Department of Pathologic Services Federal Medical Centre, Ebute‐Metta Lagos Lagos State Nigeria; ^4^ Department of Oral and Maxillofacial Surgery Obafemi Awolowo University Teaching Hospitals Complex Ile‐Ife Osun State Nigeria; ^5^ Department of Oral Pathology College of Medicine, University of Ibadan Ibadan Oyo State Nigeria; ^6^ School of Dentistry University of Rwanda Kigali Rwanda; ^7^ Faculty of Dentistry University of Puthisastra Phnom Penh Cambodia; ^8^ Department of Ophthalmology State Specialist Hospital Osogbo Osun State Nigeria; ^9^ Department of Oral Biology Rutgers School of Dental Medicine Newark New Jersey USA

**Keywords:** Gardner syndrome, impacted teeth, intestinal polyposis, jaw swelling, osteoma

## Abstract

Gardner's syndrome with the complete manifestation of colonic and extracolonic features is uncommon. Therefore, every clinician should view extracolonic features with a high index of suspicion. This may be key to early diagnosis, definitive management in these patients and importantly, helps prevent malignant transformation of existing colonic polyps.

## 1 INTRODUCTION

Gardner syndrome (GS) was first discovered by a geneticist, Eldon J. Gardner in 1951.^1^ It is a phenotypic variant of familial adenomatous polyposis (FAP) which is a rare genetic disorder with primary manifestation in the colon.^1^ There are also extracolonic manifestations of the syndrome which present as tumors and abnormalities in other parts of the body.^1^ GS is inherited in an autosomal dominant manner.^1^ It is linked genetically to the adenomatous polyposis coli (APC) gene mutation, a tumor suppressor gene located on chromosome 5q21‐22.^2^ This genetic mutation could be responsible for the wide spectrum of abnormalities seen in this condition.^3^ Colonic manifestations comprise numerous polyps in the colon and rectum while the extracolonic manifestations include: osteoma (long bone and jaw bone), dental abnormalities (hypodontia, compound odontoma, abnormal tooth morphology, supernumerary teeth, hypercementosis, dentigerous cyst, and unerupted/impacted teeth), lipoma, desmoid tumor, and congenital abnormalities (epidermoid cyst, retinal pigment change, and congenital hypertrophy of the retinal pigment epithelium (CHRPE)).^3^, ^4^, ^5^

The role of an oral and maxillofacial surgeon in the early diagnosis and referral for multidisciplinary management of GS is well documented.^2^, ^3^, ^4^ The presence of any of the dentofacial extracolonic feature(s) should raise the index of suspicion of GS.^4^ This would necessitate a diagnostic workup by a gastroenterologist. Treatment is aimed at addressing the clinical manifestations which entail surgical excision of the polyp and the extracolonic tumors. In severe cases, prophylactic colectomy to reduce the risk of malignant transformation to colonic cancer is typically performed.^6^ In this report, the authors present the first case of GS in a Nigerian adult male with the classical intestinal and extraintestinal characteristics, as well as a full histological description of intestinal samples.

## 2 CASE REPORT

### 2.1 Case history and examination

A 41‐year‐old male with no previous history of any systemic illness presented at the dental clinic of the Federal Medical Centre, Ebute‐Metta, Lagos, Nigeria, with a complaint of painful swelling at the left angle of the mandible of 8 months duration. The pain was said to worsen on lying down. Patient had a history of herniorrhaphy for inguinoscrotal hernia 1 year prior to presentation. A family history of abdominal diseases was not declared.

**FIGURE 1:**
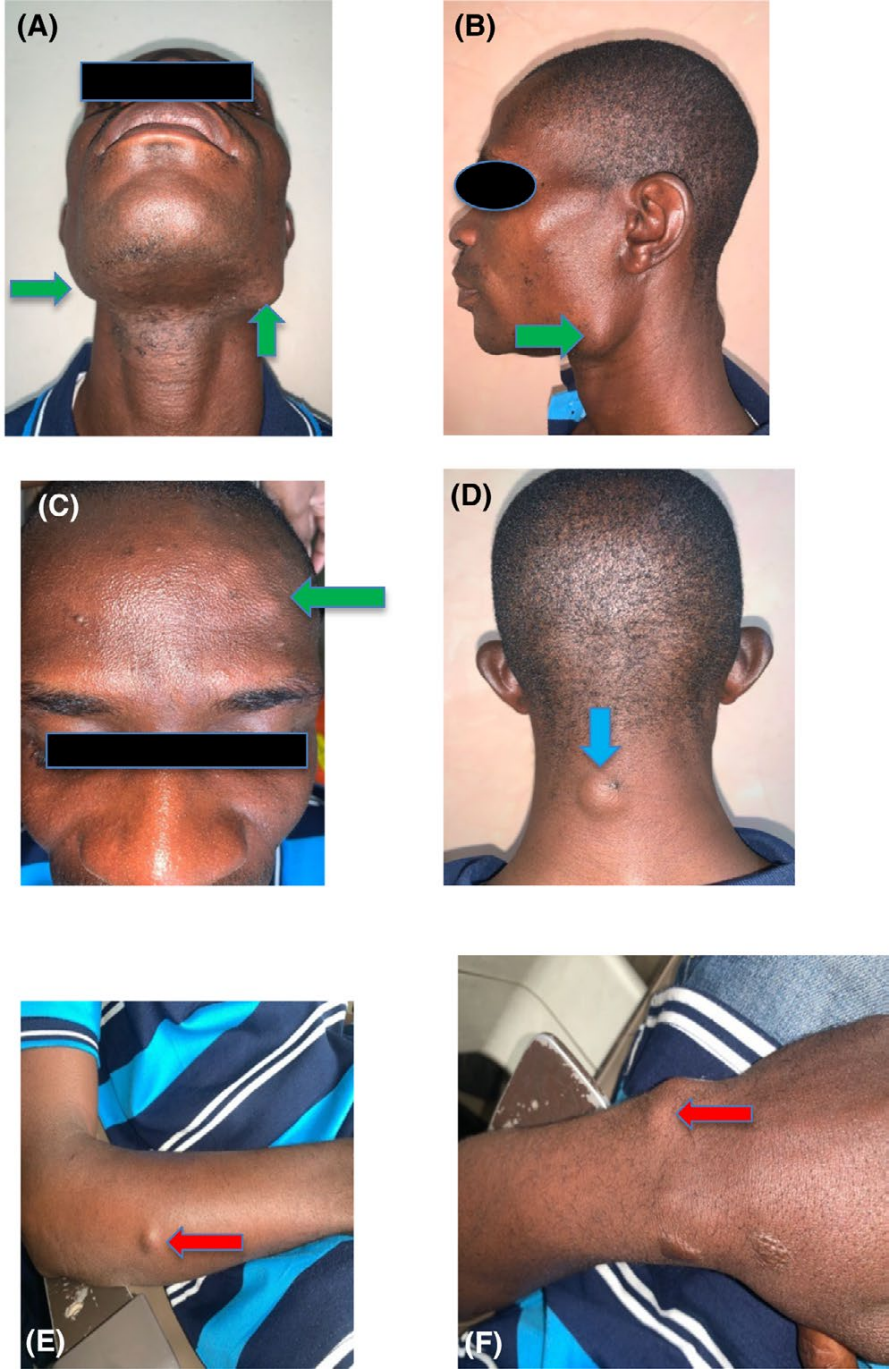
(A–C) show mandibular and frontal bone osteomata (green arrow), (D) show epidermoid cyst at the posterior aspect of the neck (blue arrow) and (E) and (F) show desmoid tumor (red arrows).

Preliminary examinations revealed a middle‐aged man with a 3 cm by 2 cm single, solitary, bony hard and slightly tender swelling at the left angle area of the mandible, unattached to the overlying skin. A similar swelling was also discovered at the right angle of the mandible, and measured approximately 0.6 cm in its widest diameter. In addition, there were multiple bony swellings on the forehead, and metachronous soft mobile subcutaneous swellings in the midline of the posterior neck, upper limbs, and gluteal area suspected to be epidermoid cysts and desmoid tumors, all appearing at different times. The submandibular lymph nodes were clinically normal. Physical examination findings are shown in Figure 1.

**FIGURE 2:**
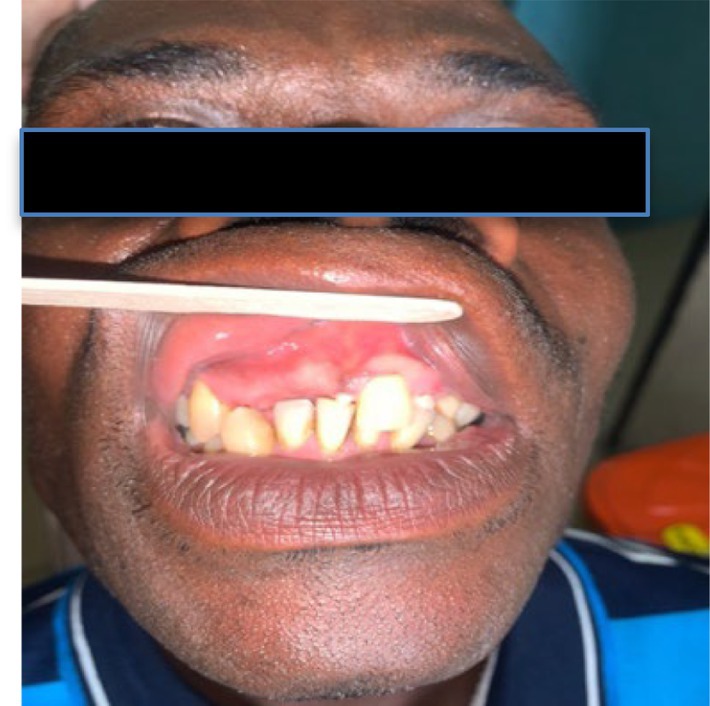
Clinical photograph multiple dental abnormalities (including missing teeth and mesiodens).

Intraoral examination revealed a mesiodens, retained upper left deciduous canine, missing upper right first incisor, upper left second incisor, and canine (Figure 2). The lower left third molar was covered by inflamed and tender pericoronal flap.

### 2.2 Diagnosis, investigations and intervention

**FIGURE 3:**
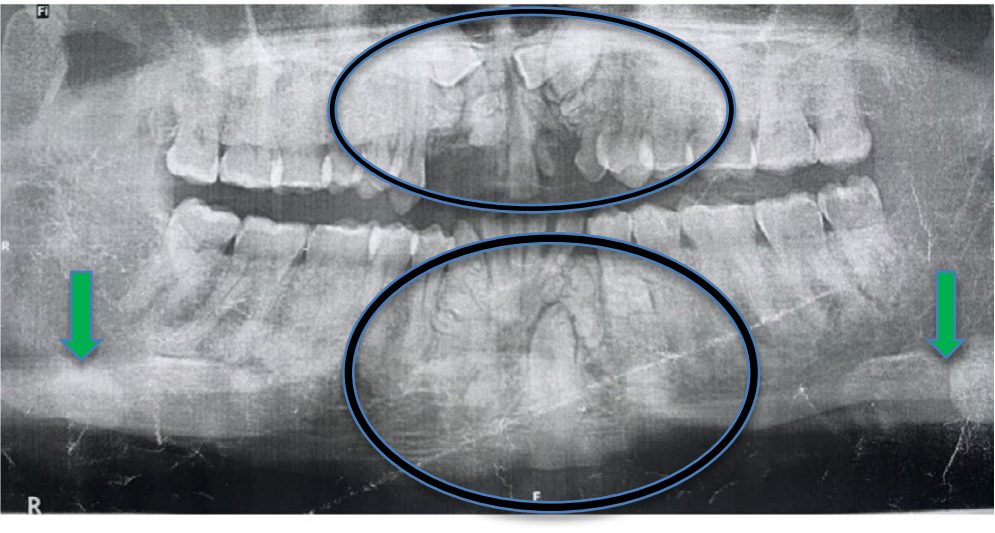
Panoramic radiograph showing mandibular osteomas (green arrows), multiple dental abnormalities including multiple impacted teeth, supernumeraries, and odontomas (circled).

Based on the presenting clinical features, panoramic radiograph was ordered. This showed multiple radiopaque elements suggestive of multiple impacted teeth, multiple odontoma, and osteomas (Figure 3). GS was suspected based on the presenting clinical and radiographic symptoms notwithstanding the absence of abdominal complaints and normal hematological test results.

**FIGURE 4:**
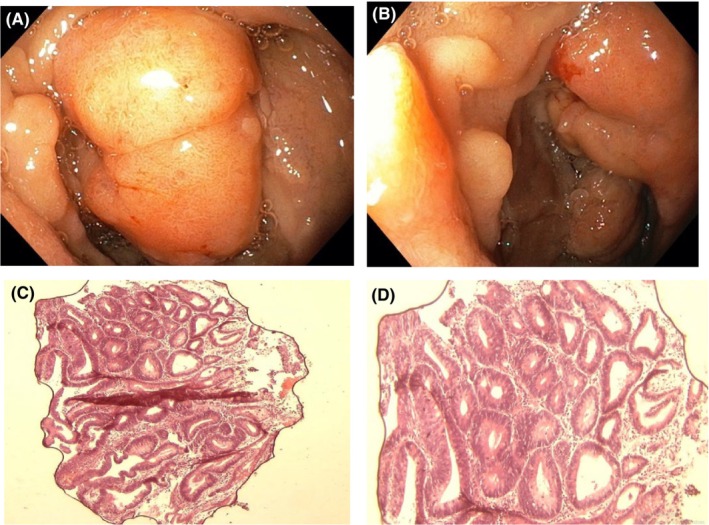
(A) and (B) show endoscopic images of duodenal polyp; (C) and (D) are photomicrographs of duodenal polyps at X40 and X100, respectively.

**FIGURE 5:**
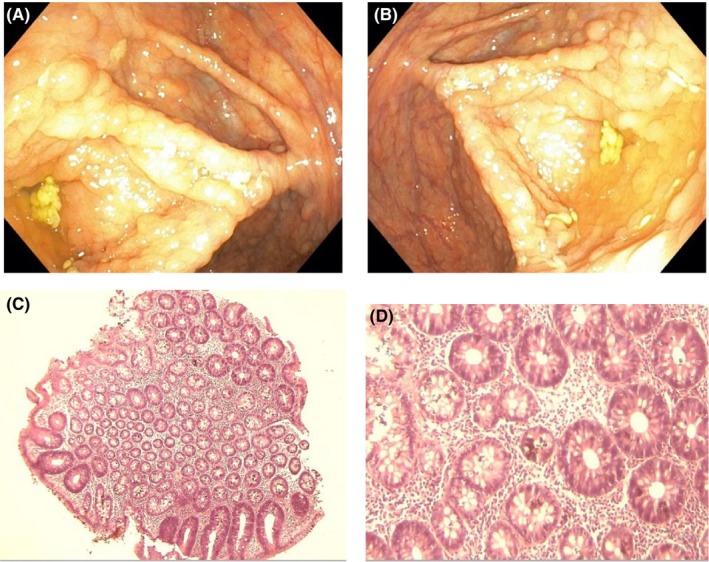
(A) and (B) are the endoscopic images of caecal polyps while (C) and (D) are histological sections of caecal polyp at X40 and X100, respectively.

**FIGURE 6:**
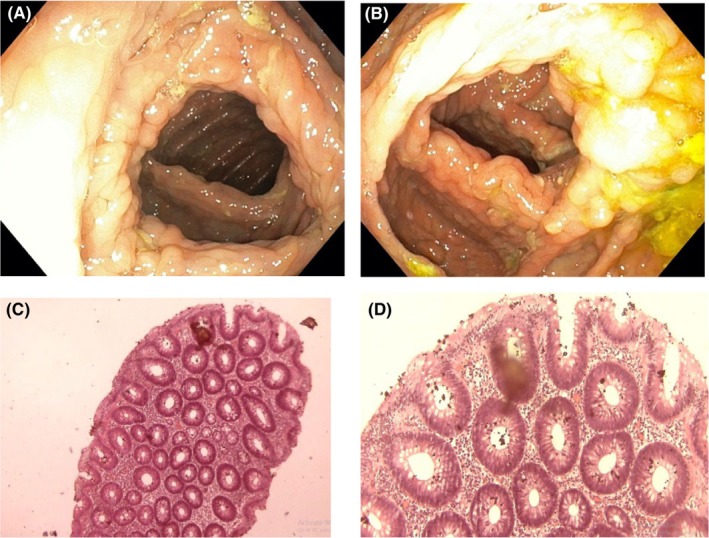
(A) and (B) are the endoscopic images of the transverse colon. (C) and (D) show the histological sections of the transverse colonic polyp at X40 and X100, respectively.

**FIGURE 7:**
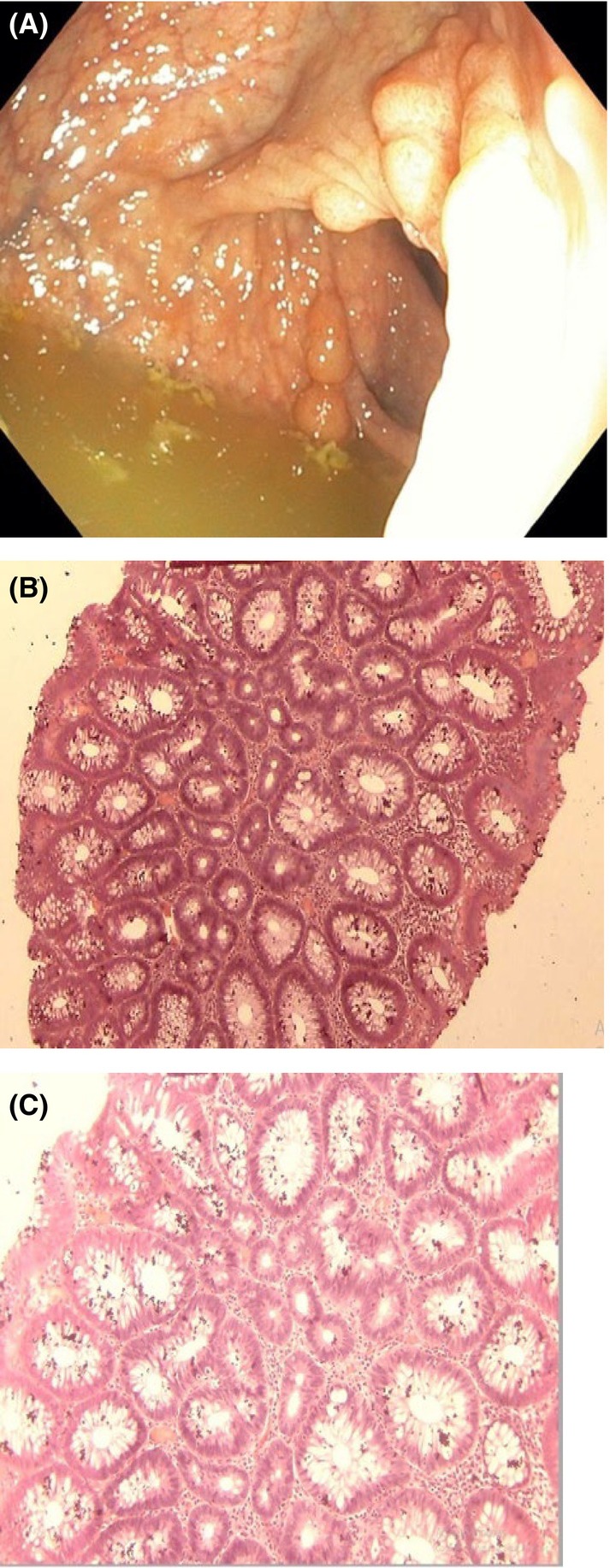
(A) shows the endoscopic image of the sigmoid polyp. (B) and (C) are photomicrographs of the sigmoid polyp at X40 and X100 magnifications, respectively.

**FIGURE 8:**
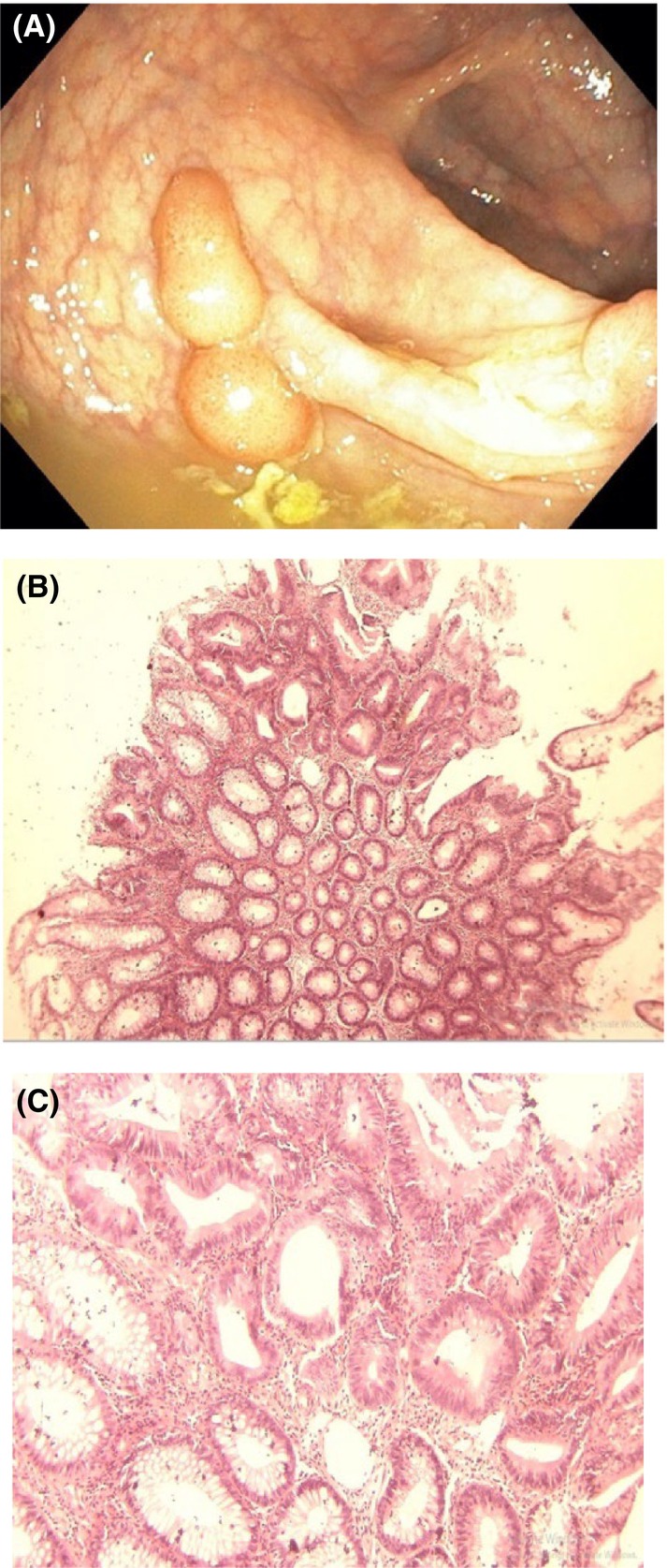
(A) shows the endoscopic view of the rectal polyps. (B) and (C) are photomicrographs of the rectal polyps at X40 and X100, respectively.

Gastrointestinal endoscopy was ordered to rule out the presence of intestinal polyposis. This was done via the oral cavity and anus using a flexible Olympus endoscope under direct visualization. Endoscopy revealed multiple sessile and pedunculated polyps in the second part of the duodenum (Figure 4A,B), caecum (Figure 5A,B), transverse colon (Figure 6A,B), sigmoid colon (Figure 7A), and multiple polyps in the rectum (Figure 8A), all estimated to be more than one hundred in number. Cold forceps biopsy was done from multiple polyps and hemostasis was ensured. Histological evaluation of the multiple intestinal polyps showed varying degrees of dysplasia as follows:

Figure 4C,D show the histological sections of the duodenal polyp. There is a polypoid fragment of tissue comprising of crypts and mucosal glands lined by dysplastic epithelium having hyperchromatic nuclei with nuclear spindling and focal nuclear stratification extending up to the luminal surface in areas. In addition, there is focal loss of basal polarity, consistent with tubular adenoma with high‐grade dysplasia.

Figure 5C,D show the histological sections of the caecal polyp exhibiting low‐grade dysplasia of the surface epithelium.

Figure 6C,D show the histological section of the transverse colonic polyp exhibiting features consistent with tubular adenoma with low‐grade dysplasia.

Figure 7B,C show the histological sections of the sigmoid polyp. The histological sections reveal focal areas of dysplastic colonic epithelium having hyperchromatic nuclei with nuclear spindling, focal nuclear stratification limited to the lower third and loss of polarity. Some of the apical glands show slight reduction of mucin content and the lamina propria appear moderately infiltrated by chronic inflammatory cells in keeping with tubular adenoma with low‐grade dysplasia.

Figure 8B,C show histological sections of the rectal polyp showing features that are consistent with tubular adenoma with low‐grade dysplasia.

A definitive diagnosis of Gardner syndrome was made based on the first diagnostic criteria of the World Health Organization.^7^ Based on this, the patient was educated about the risks associated with the intestinal findings, and was counseled about the need for screening of family members. The high possibility of malignant transformation was explained to the patient. Surgical interventions including polypectomy and colonic resection, as well as the likely complications were also discussed as part of counseling. He later had transalveolar extraction of the lower left third molar and there has been no recurrence of jaw pain in the preceding 6 months. The patient refused involvement of his family and declined treatment of abdominal diseases citing fears of perceived postsurgical complications. He opted to wait and see. The patient is on a three‐monthly follow‐up review and no abdominal symptom is reported yet.

## 3 DISCUSSION

The dental patient presented in this report was identified with the classic clinical characteristics of GS through a high index of clinical suspicion, followed by confirmatory inquiry into the World Health Organization's criteria for diagnosis.^7^ The patient presented at the dental clinic with the complaint of a painful swelling at the angle of the mandible. He was oblivious to the presence of other smaller swellings present on the other side of the jaw and face possibly due to lack of pain. During routine dental examination, a cluster of other dental abnormalities such as missing and malformed teeth, odontomes, and jaw osteoma were discovered. This gave a clue for the probability of a more generalized clinical condition.

Orofacial disorders are often part of a spectrum of more serious systemic diseases.^8^, ^9^, ^10^ Sometimes, the oral lesions occur before the onset of systemic disease. In other situations, oral lesions may occur simultaneously or after the disease has resolved.^8^ These lesions may therefore be useful in the development of differential diagnoses of systemic conditions, offer an easy biopsy site and in other circumstances, provides adjuncts for evaluating disease severity and prognosis.^8^, ^11^

Gardner syndrome is a typical example of such diseases with typical but under‐reported orofacial disorders. It is a cluster of pathologies caused by a genetic defect on chromosome 5.^8^ It was previously considered as a distinct entity from familial adenomatous polyposis but is now recognized as part of the disease spectrum involving defects in the derivatives of all the three primordial germ layers.^12^, ^13^ It is characterized by high‐risk intestinal polyposis and a number of extracolonic changes involving various organ‐systems.^8^, ^14^ The head and neck manifestations often begin in childhood or adolescence and may include multiple jaw enostoses, supernumerary, and/or unerupted teeth, increased risk of odontomas, jaw and paranasal sinus osteomas, and epidermoid cysts of the skin of head and neck.^8^, ^15^

Oftentimes, extracolonic manifestations of GS are subclinical,^12^ and this may be responsible for late presentation and delay in diagnosis and treatment. The index patient presented at the hospital on account of pain even though the jaw swelling was present much longer. Misrepresentation of symptoms, lack of pain, the belief that symptoms will resolve on their own, self‐medication, financial constraints, and lukewarm attitude have been identified as some reasons for late hospital presentation.^16^, ^17^, ^18^ Frequently, diagnostic delay is identified as a reason for late presentation to the specialist. The quality of clinical reasoning and oftentimes, diagnostic difficulty are drivers of missed diagnosis and delayed referrals to specialists in many clinical settings.^19^ In this review, the genetic aspect and clinical presentations of GS are revisited as clusters of symptoms and signs identified in this patient are not uncommon presentations in secondary and tertiary health institutions.

### 3.1 The genetic aspect of Gardner syndrome

Gardner syndrome is an autosomal dominant disorder with almost 80% penetrance and variable expressivity.^20^, ^21^ It is caused by truncating mutations of band 5q21‐q22 which produces a multidomain multifunctional protein involved in wnt signaling and microtubule function.^15^, ^21^, ^22^ This mutation causes functional inactivation of the tumor suppressor activity of the adenomatous polyposis coli (APC) gene.^15^, ^21^, ^23^

There are uncertainties about the exact breakpoint on the 5q and a study reported a variation from q13 to 31.^24^ Over 1400 different mutations of this gene have been reported, and the specific area of the APC gene affected determines the extracolonic presentation as well as the number, time frame, and the malignant potential of the intestinal polyp.^21^, ^25^ For instance, mutation at codon 1309 is associated with early onset of polyposis and cancer whereas mutation at the extreme 5′ end of the same gene is associated with attenuated phenotype.^25^ Even for identical mutations, phenotypic expressions differ.^15^, ^22^

### 3.2 Epidemiology

The global incidence of GS is unknown but the prevalence varies between 1 in 12,000 and 1 in 4000 depending on the region and the knowledge of the variation in the expression of GS phenotype.^25^, ^26^, ^27^, ^28^ In Nigeria, there is currently no available data about the incidence of GS in the literature. William et al., however, showed that the average age of Nigerians with intestinal polyps was lower than the average age at which premalignant intestinal polyp usually develop, with an observed male predilection.^29^, ^30^ When penetrance is less marked or there are multiple mutations of different but closely linked dominant genes, the associated clinical characteristics may vary. This may be responsible for the wide variation in the syndrome reported in the literature.^31^ Until recently, previous reports have described the syndrome as a distinct entity from FAP,^22^ and less than a third of all cases of familial polyposis are thought to be GS.^32^ Available evidences do not suggest a racial or gender predilection of GS.^33^

### 3.3 Gastrointestinal presentations

Gardner syndrome is a triad of multiple intestinal polyposis, soft tissue tumors, and hard tissue tumors.^34^ Gastrointestinal polyps, especially in the colon and rectum, are clinically significant because of high risk of malignant transformation.^34^, ^35^ The polyps may be solitary, and this usually arises sporadically.^36^ Its incidence increases with age and is found in about a tenth of the adult population.^36^

The period of life at which the polyposis first appears is controversial.^37^ Some investigators opine that polyps develop in young adulthood while others consider the possibility of presentation earlier in life, or even at birth.^37^, ^38^ Colorectal polyps are often visible at puberty but remain undiagnosed until the third decade of life. In many situations, anemia, constipation, bloody diarrhea, bowel obstruction, mucous discharge, cramping, and abdominal pain are the first symptoms that patients present with.^21^, ^35^, ^38^, ^39^, ^40^

In this reported case, the patient presented without abdominal symptoms despite the presence of extensive diffuse polyps in the second part of the duodenum, colon, caecum, and rectum. This is similar to the findings by Panjwani et al.,^39^ Boffano et al.^35^

### 3.4 Risk of malignant transformation of intestinal polyps

Untreated polyposis of GS has a 100% risk of undergoing malignant transformation at about the fourth decade of life in many reported cases.^15^, ^35^, ^38^, ^41^, ^42^, ^43^ Coli et al.^43^ reported that the average age at death for colon cancer in GS was 41 years. This is over 25 years earlier than the age of death from isolated colon cancer in the general population.^43^ Overall, the risk of periampullary carcinoma in patients with GS is 300 times higher when compared with the general population.^44^

At the time of this report, no clinical sign of malignant transformation is obvious in our patient. Although the patient is 41 years of age and no immediate signs of malignancy was noted, significant dysplastic changes were noted on histology of biopsied intestinal polyps.

### 3.5 Extraintestinal presentations

Osteomas, epidermoid cysts, and subcutaneous fibromas, along with multiple adenomas formed the basis of the disease entity named Gardner syndrome by Smith in 1958.^36^, ^45^ The dental features including unerupted and supernumerary teeth, and odontomas were added by Fader et al. in 1962.^46^ The dental characteristics found in the syndrome have been described as being secondary to osteomas or other causes^36^ Oftentimes, maxillofacial features are the first to appear.^31^, ^37^, ^47^

More than 90% incidence of osteoma is reported in patients with GS, and the average number of bone lesions ranges between 2.9 and 4.7.^35^ It is more common in females, predominantly in the second and third decades of life.^35^ The mandible is the most common site for osteoma followed by the frontal bone, and the molar‐ramus area is the most frequently affected in the mandible.^48^ In this case, there was a disfiguring osteoma in the left molar‐ramus area of the mandible while the right molar‐ramus area of the mandible and frontal bone were affected by multiple, but smaller osteomata.

The most frequent soft tissue pathology in GS is epidermoid inclusion cyst and it is usually found on the face or in the extremities.^49^ Desmoid tumors are found in up to 17%, and can be as high as 66% of patients often occurring at incision sites, the abdominal cavity, or the retroperitoneum.^21^, ^35^, ^49^

Dental abnormalities such as single or multiple supernumerary teeth, odontoma, missing and unerupted teeth, and hypercementosis have been reported in more than 50% of patients and are, oftentimes, the first clinical features in the diagnostic process.^35^, ^39^, ^49^

Nine out of 10 patients with GS presents with hypertrophy of the retinal pigmented layer according to a report.^21^ This is rarely symptomatic and are often discovered incidentally during routine eye examination.^50^

The authors found multiple osteomata, epidermoid cysts, multiple dental abnormalities as well as intestinal polyposis in the index patient. No history of rectal bleed or bowel perforation was found on colonoscopy.

### 3.6 Diagnostic criteria

Although primarily based on clinical findings, the diagnosis of GS can be difficult due to high variation in the extraintestinal features.^42^ In such situations, genetic testing for mutations or demonstration of multiple colonic polyps on colonoscopy is usually confirmatory.^21^

Duncan et al.^38^ proposed either of two diagnostic criteria for GS: at least two manifestations of the diagnostic triad (intestinal polyps, soft tissue tumors, and hard tissue tumors), or one manifestation and a blood relative with at least two of the clinical manifestations without the inclusion of FAP.^38^ Another set of diagnostic criteria was proposed by Pauli et al.^32^ viz (1) the presence of the primary triad (colonic polyps, soft tissue tumors, and osteomas) in a single patient, or (2) the presence of any of the usual findings in an individual with a family member who has all the three primary findings or who is from a family in which various members collectively have all three primary findings.

However, the WHO listed three clinical scenarios as diagnostic criteria for GS (1) 100 or more colorectal polyposis; (2) APC gene germline mutation; (3) a family history of FAP, and at least one epidermoid cyst, osteoma or desmoid tumor.^7^, ^51^ The patient met the first and third criteria of the WHO, as well as the first criteria of Duncan et al. and Pauli et al.^38^

### 3.7 Screening and treatment

Patients with GS, or their relatives should undergo surveillance programs including, large bowel and upper gastrointestinal tract surveillance.^21^ It is advocated that genetic risk testing should precede the initiation of regular endoscopic testing.^15^ Large bowel surveillance includes annual flexible sigmoidoscopy starting from age 13 till 30 in patients with a family history of FAP, but who has no demonstrable mutation. This should be repeated every 3–5 years from age 30 to 60 years.^21^ Where there is known mutation of the APC gene, flexible sigmoidoscopy every 6 months, and annual colonoscopy should be done starting from the age of 10. Such patients should be counseled on the need for surgery before the age of 25.^21^

From the oral surgeon's point of view, osteomata, and multiple dental abnormalities should raise a suspicion. Simple imaging such as panoramic radiograph and advance imaging like craniofacial computed tomography (CT) scan may provide screening methods for more sinister abnormalities and early diagnoses.^21^, ^39^, ^52^ Ophthalmologist also have important roles to play as the presence of characteristic pigmented fundus lesions occur in up to 80% of patients with FAP, and is often present at birth, preceding the development of intestinal polyposis.^53^

The treatment of GS is patient‐specific and the intervention for extraintestinal pathologies are often symptomatic. These range from simple observation to cosmetic excision of disfiguring osteomata especially when it interferes with function, extraction of impacted teeth, cyst enucleation, dental prosthesis, and so on.^44^

Restorative proctocolectomy with ileal pouch‐anal anastomosis (RPC‐IPAA) is the best surgical intervention for FAP (including GS) patients because it resects all intestinal mucous membrane to avoid malignant transformation and still preserves intestinal functions.^54^ This approach avoids colostomy, thus improving patient's quality of life (QoL).^54^ Our patient declined surgical intervention for intestinal polyp based on concerns about his quality of life in the postoperative period. Financial access to care was also a major impediment to his care, a significant aspect of which was borne by the hospital.

A high index of clinical suspicion of synchronous jaw and dental abnormalities should point to high‐risk systemic conditions thereby assisting with early identification and referral to appropriate specialist. It is important that clinicians, especially dentists, are familiar with the maxillofacial and dermatological features of Gardner Syndrome since they often precede intestinal polyposis.

## AUTHOR CONTRIBUTIONS

**Olusegun I. Olaopa:** Conceptualization; data curation; formal analysis; funding acquisition; investigation; methodology; project administration; resources; software; supervision; validation; visualization; writing – original draft; writing – review and editing. **Adedamola A. Dada:** Data curation; investigation; resources; writing – original draft; writing – review and editing. **Oluwafunmilayo Y. Soneye:** Data curation; investigation; resources; writing – review and editing. **Oluwadamilare Iyapo:** Data curation; investigation; resources; writing – original draft; writing – review and editing. **Taofeek A. Akinniyi:** Writing – review and editing. **Akinyele O. Adisa:** Writing – review and editing. **Kehinde K. Kanmodi:** Resources; writing – review and editing. **Adedolapo O. Olaopa:** Resources; writing – review and editing. **Christian I. Emeka:** Writing – review and editing. **Imudia D. Ehanire:** Writing – review and editing. **Modupe Coker:** Writing – review and editing.

## FUNDING INFORMATION

No funding was received for the study.

## CONFLICT OF INTEREST STATEMENT

Authors have no competing interest to declare.

## CONSENT

Written informed consent was obtained from the patient to publish this report in accordance with the journal's patient consent policy.

## CONSENT TO PARTICIPATE

Informed consent about clinical management was obtained from the patient in this case.

## Data Availability

The authors confirm that the data supporting the findings of this study is available upon request from the corresponding author.
